# Assessment of teachers’ knowledge, awareness, and response to students with asthma in Greater Cairo schools

**DOI:** 10.1038/s41533-026-00571-w

**Published:** 2026-10-23

**Authors:** Hoda M. Abdel-Hamid, Radwa M. Elsayed, Sara El-Sayed Abd El-Ghani, Marwa M. Zein, Hebatallah M. Hassaan, Abdelhameed Elshenawy, Mostafa Alaa Abdelmoneim, Jumana Hesham Ismail, Fatma Elzahraa Ahmed Abd Elaleem, Hadeel Abd El Wahab Mohamed

**Affiliations:** 1 Department of Chest Diseases, Faculty of Medicine, Cairo University, Cairo, Egypt; 2 Department of Family Medicine, Faculty of Medicine, Cairo University, Cairo, Egypt; 3 Department of Internal Medicine, Clinical Hematology unit, Faculty of Medicine, Cairo University, Cairo, Egypt; 4 Department of Public Health and Community Medicine, Faculty of Medicine, Cairo University, Cairo, Egypt; 5 Department of Pediatrics, Faculty of Medicine, Cairo University, Cairo, Egypt; 6 Department of Critical Care and Emergency Nursing, Faculty of Nursing, Cairo University, Cairo, Egypt; 7 Intern, Faculty of Medicine, Cairo University, Cairo, Egypt

**Keywords:** Diseases, Health care, Medical research

## Abstract

Bronchial asthma remains a significant public health challenge, especially among school-aged children. Schoolteachers are often the initial responders to asthma attacks, making their role vital for early symptom recognition and first aid provision. Therefore, this study aimed to explore the knowledge, awareness, and response of schoolteachers to students with asthma. Between March 2024 and October 2024, a self-administered questionnaire was created on Google Forms and distributed to schoolteachers through WhatsApp groups of private and public schools across different districts in Greater Cairo. The questionnaire consisted of three sections assessing: (1) teachers’ experience and demographics, (2) basic knowledge of asthma, and (3) knowledge related to first aid response in an asthma attack. A total of 634 schoolteachers participated in the study. Of these, 361 (57%) reported having previously taught students with bronchial asthma, whereas only 176 (27.8%) had witnessed a student having an acute asthma attack before. A majority of participants (445/634, 70.2%) correctly identified the short-acting beta agonist (SABA) inhaler or nebulizer as appropriate first aid for an acute asthma attack. However, only 187/634 (29.5%) reported knowing the correct technique for using SABA inhalers or nebulizers during an asthma attack. More than half of the participants, 362/634 (57.1%), provided appropriate answers regarding the etiology of the disease, specifically whether it was a contagious disease or not. There was no statistically significant association between teachers’ knowledge level and their sociodemographic characteristics. Teachers in Greater Cairo demonstrated fair knowledge and awareness regarding asthma. However, they did not fully understand appropriate first-aid responses, a gap that underscores the need for more asthma education programs.

## Introduction

Asthma is the most prevalent chronic pediatric illness and the leading cause of childhood morbidity from chronic disease as measured by school absences, emergency department visits, and hospitalization^1^. It accounts for more school absences than any other pediatric condition, with an average of 9.7 absence days annually. This prolonged absence interferes with the learning process and has an impact on a child’s academic achievement^2^.

The proper management of asthma in schools depends on asthma awareness, the observation of asthma symptoms, the identification of possible asthma attacks, and the application of the required therapy. Asthma management should be optimized in the school setting due to the large number of asthmatic children and the fact that children spend much of their time in school^3^. Also, schools contain a variety of allergens, including dust, fumes, chalk dust, and paper; these were identified as common triggers of asthma symptoms that may affect the incidence of asthma in schoolchildren^4^.

Additionally, exercise and increased activity are major triggers for many asthmatic children. Cough, dyspnea, and sometimes wheezing are signs of exercise-induced asthma, which occur after prolonged rapid breathing, such as after running. Therefore, it is advisable to discuss this with the gym instructor during parent-teacher meetings^5^.

The Global Initiative for Asthma (GINA, 2026) emphasizes that effective childhood asthma care relies on school-based management^6^. The Centers for Disease Control and Prevention (CDC) Managing Asthma in Schools Guidelines outline six core strategies to help students manage their conditions. These involve establishing support systems, providing health services, offering education, reducing indoor/outdoor triggers, adapting physical education, and coordinating efforts between the school, family, and medical providers^7^.

Globally, the incidence and prevalence of asthma among young children range between 8% and 28%, with figures increasing annually^8^. In Egypt, bronchial asthma represents a significant public health challenge. Studies across various regions indicate a prevalence of 4.8% in infants and children under four years old, 7.7% in the Nile Delta region^9^, and as high as 9.4% among adolescents in Cairo^10^.

As a megacity, the Greater Cairo Governorate offers a large and diverse sample of socioeconomic and educational environments. It includes both public and private schools, allowing for broader representation. Additionally, it presents unique environmental stressors such as high levels of air pollution and classroom overcrowding^11,12^.

A significant gap remains in the current Egyptian literature. Without understanding the current baseline of teacher knowledge in this high-density region, it is impossible to implement targeted educational interventions that could mitigate the academic “ achievement gap “ faced by asthmatic children.

Therefore, the present study was conducted to address this deficiency by evaluating Greater Cairo school teachers’ knowledge and awareness of asthma, in addition to assessing teachers’ familiarity with school-based emergency response protocols.

## Methods

### Study design and participants

This cross-sectional study was conducted in private and public schools across the Greater Cairo governorate, covering its different districts, between March 2024 and October 2024. The purpose of the study was explained to heads of educational directorates to obtain their permission and to distribute an online survey that was created using Google Forms to assess teachers’ awareness, knowledge, and first aid response to students with asthma.

### Sampling technique

A convenience sampling technique was used. The survey was distributed electronically via schools’ WhatsApp groups across 16 private and public schools in Greater Cairo, comprising 925 teachers. A reminder was sent two weeks later to increase the response rate and ensure participation from teachers who had not yet responded. The response rate in this study was 68.5%.

### Sample size

The sample size of 535 was calculated using OpenEpi (www.openepi.com), based on an anticipated 60% prevalence of knowledge of asthma disease management among schoolteachers from a previous study conducted in Saudi Arabia^2^, with a 95% confidence interval, 80% study power, and a 35% assumed non-response rate^13^.

$${{{\rm{Equation}}}}:{{{\rm{Sample}}}} \, {{{\rm{size}}}} \, {{{\rm{n}}}} = 	[{{{\rm{DEFF}}}} * {{{\rm{Np}}}}(1-{{{\rm{p}}}})]/ \\ 	 \left[\left({{{\rm{d}}}}2/{{{\rm{Z}}}}21-{{{\rm{\alpha }}}}/2 * ({{{\rm{N}}}}-1) \right. \right. \\ 	 \left. + {{{\rm{p}}}} * (1-{{{\rm{p}}}})\right]$$

### Questionnaire development and data collection

Teachers were asked to complete a self-administered, three-section online questionnaire to collect data. Most of the questions were adapted from previous studies^2,14^. The questionnaire was translated into Arabic by two independent translators and then back-translated into English to ensure accuracy and consistency. Content validity was assessed by a panel of three experts in pulmonology, epidemiology, and public health, who reviewed the questionnaire for relevance, clarity, and comprehensiveness.

Construct validity assessment, including exploratory or confirmatory factor analysis, was not performed, as the questionnaire was adapted from previously published instruments rather than newly developed.

A pilot study was conducted on 15 schoolteachers who were not included in the final analysis to evaluate the clarity and applicability of the questionnaire. Minor modifications were made based on their feedback.

The internal consistency of the questionnaire was assessed using Cronbach’s alpha coefficient, which demonstrated acceptable reliability (α = 0.753).

Informed consent and written instructions on how to fill out the questionnaire were included in the first section of the survey, which was followed by inquiries regarding the participants’: 1) teaching experience and demographics; 2) basic knowledge and awareness of asthma (including the nature of the disease, symptoms of an asthma attack, and symptoms that signify worsening of an asthma attack). Additionally, questions explored the teachers’ knowledge of asthma triggers (chalk dust, dusty weather, cold weather, passive smoking, and physical exercise); and 3) knowledge related to first aid response in an acute asthma attack. These questions assessed the teachers’ response to first-aid measures needed for an asthma attack, the proper technique for using inhalers, nebulizers, and spacer devices during an attack, and the expected time after inhaler use to relieve an asthma attack. The questionnaire also evaluated common misconceptions regarding asthma and teachers’ readiness to take part in an educational program.

### Data quality

The questionnaire was administered using Google Forms. To enhance data quality, the survey was configured to allow only one response per participant. In addition, all questionnaire items were set as mandatory; therefore, questionnaires could not be submitted unless all questions had been completed. Consequently, there were no missing data in the final dataset.

### Ethical considerations

The study was carried out in accordance with the Declaration of Helsinki, and each participant gave informed consent. Cairo University’s Research and Ethics Committee reviewed and approved this work (ethical permission number 59-2024).

### Statistical analysis

For logical consistency and completeness, all collected data were reviewed. Pre-coded data were retrieved from the Google Form and then imported into the Statistical Package for the Social Sciences (SPSS), version 23, for statistical analysis. The mean and standard deviation were used to summarize the data for quantitative variables. Frequency and percentage were used to summarize the data for qualitative variables.

The knowledge score was calculated for 32 knowledge questions, and the percent score was then calculated. Each correct response in the knowledge section was assigned a score of 1, while incorrect or **“** don’t know **“** responses were scored as **0**. The total knowledge score ranged from 0 to 32. A percentage score was calculated and categorized as poor (<50%), fair (50–65%), and good (>65%). These cut-off values were adopted from previous literature to facilitate comparison with similar studies^15^ to facilitate comparison with similar studies. They were used as operational categories for interpreting knowledge levels rather than representing independently validated thresholds. Associations between categorical variables and knowledge score categories were assessed using the chi-square test, while comparisons of mean knowledge scores across groups were performed using one-way ANOVA, as appropriate. The p-value of ≤ 0.05 was considered significant. The continuous knowledge score (range: 0–32) was used as the dependent variable in the multivariable regression analysis. Although the knowledge score was categorized into poor, fair, and good levels for descriptive purposes, the continuous score was retained for regression analysis to preserve information and maximize statistical power. A multivariable linear regression model was used to identify independent predictors of the teachers’ knowledge. Regression results were reported as beta coefficients with corresponding 95% confidence intervals (CIs) and p-values.

### Ethics approval and consent to participate

The study was conducted in accordance with the Declaration of Helsinki, and each participant provided informed consent. Cairo University’s Research and Ethics Committee reviewed and approved this work (ethical permission number 59-2024).

## Results

A total of 634 teachers responded to the questionnaire. Their mean age was 40 ± 10 years, and 211/634 (33.3%) had 20 years or more of teaching experience. The thorough sociodemographic characteristics of the respondents are presented in Table 1.

**Table 1:** Sociodemographic characteristics of the respondents.

Respondents (*n* = 634)		
Age (mean ± SD)	40 ± 10	
Gender *n* (%)	Males	139 (21.9%)
	Females	495 (78.1%)
Years of teaching experience *n* (%)	1–4 years	115 (18.1%)
	5–9 years	103 (16.2%)
	10–14 years	137 (21.6%)
	15–19 years	68 (10.7%)
	≥20 years	211 (33.3%)
School type *n* (%)	Public	305 (48.1%)
	Private	329 (51.9%)
Family or relative history of asthma *n* (%)	Yes	261 (41.2%)
	No	373 (58.8%)
How many students have you dealt with who suffered from bronchial asthma? *n* (%)	<5 students	225 (35.5%)
	5–10 students	76 (12%)
	> 10 students	60 (9.5%)
	I have not dealt with students suffering from bronchial asthma	273 (43%)
Have you ever witnessed a student having an acute asthma attack? *n* (%)	Yes	176 (27.8%)
	No	458 (72.2%)

*SD* standard deviation.

Regarding experience with asthma, out of 634 participants, 361 (57%) reported having previously taught students with bronchial asthma, whereas only 176 (27.8%) had witnessed a student having an acute asthma attack before. Additionally, 261 (41.2%) of schoolteachers had a family history or a relative with a history of asthma.

### Basic knowledge of schoolteachers regarding asthma

More than half of the participants, 362/634 (57.1%), provided appropriate answers regarding the etiology of the disease, specifically whether it was a contagious disease or not. Table 2 shows comprehensive responses to additional questions that assessed the teachers’ asthma background knowledge.

**Table 2:** Basic knowledge of schoolteachers regarding asthma and first aid in acute asthmatic attack.

Respondents (*n* = 634)		
Is asthma a contagious disease? *n* (%)	Yes	75 (11.8%)
	No	362 (57.1%)
	I do not know	197 (31.1%)
What are the symptoms of asthma? *n* (%)	Cough	379 (59.8%)
	Sneezing and a runny nose	39 (6.2%)
	Wheezes	317 (50%)
	Dyspnea	505 (79.7%)
	Cyanosis	147 (23.2%)
	Chest pain	267 (42.1%)
	Vomiting	25 (3.9%)
	I don’t know	77 (12.1%)
Symptoms that indicate a worsening acute asthma attack *n* (%)	Irritability	156 (24.6%)
	Dizziness	27 (4.3%)
	Lip cyanosis	253 (39.9%)
	Convulsions	127 (27.8%)
	Chest pain	313 (49.4%)
	I do not know	168 (26.5%)
What is the first aid needed for a student suffering from an acute asthma attack? *n* (%)	SABA inhaler or nebulizer	445 (70.2%)
	Hospitalization	260 (41%)
	Antibiotics	31 (4.9%)
	CPR	28 (4.4%)
	Traditional /faith-based therapy	65 (10.3%)
	I do not know	7 (1.1%)
Do you know the proper technique for using SABA inhalers and spacers or nebulizers during an asthma attack? *n* (%)	Yes	187 (29.5%)
	No	447 (70.5%)
How do you know the proper technique? *n* (%) *n* = *187*	I had a family history of asthma	126 (67.3%)
	Social media	71 (38%)
	Teacher preparation program	5 (2.7%)
	Other: I have bronchial asthma	2 (1.1%)
Does your school have a nebulizer device? *n* (%)	Yes	102 (16.1%)
	No	309 (48.7%)
	Don’t Know	223 (35.2%)
Is there an oxygen cylinder in your school? *n* (%)	Yes	130 (20.5%)
	No	274 (43.2%)
	Don’t Know	230 (36.3%)
Expected time after using an inhaler to relieve a bronchial asthma attack? *n* (%)	5–10 mins	190 (30%)
	10–20 mins	145 (22.9%)
	> 30 mins	22 (3.5%)
	Don’t know	277 (43.7%)
In your opinion, does bronchial asthma negatively affect academic progress? *n* (%)	Yes	295 (46.5%)
	No	189 (29.8%)
	Don’t know	150 (23.7%)
Do you think that the family of a child suffering from bronchial asthma might be ashamed or denied mentioning the disease to his schoolteacher?	Yes	93 (14.7%)
	No	443 (69.9%)
	Don’t know	98 (15.4%)
Because you answered “yes,” Please state reasons. *n* (%) *n* = *93*	Believing that the student might be ashamed	60 (64.5%)
	Denying the existence of a health condition	20 (21.5%)
	Worrying that the student might not be accepted into school if the illness is disclosed	30 (32.3%)
	Feeling that the school has no role in monitoring the student’s health	23 (24.7%)
	Fear that the student will be maltreated by his teachers	43 (46.2%)
Do you need to know more about dealing with students with bronchial asthma? *n* (%)	Yes	512 (80.8%)
	No	122 (19.2%)

*CPR*, Cardiopulmonary resuscitation.

Of the participants, 295/634 (46.5%) believed that asthma negatively affects academic progress, and some schoolteachers (*n* = 93, 14.7%) thought that the family of a student with bronchial asthma might be embarrassed or hesitant to mention the condition to their schoolteacher. These teachers cited the following reasons: believing the student might be ashamed (*n* = 60, 64.5%), denial of having the disease (*n* = 20, 21.5%), worrying that the student might not be accepted into school if the illness is disclosed (*n* = 30, 32.3%), feeling that the school has no role in monitoring the student’s health (*n* = 23, 24.7%), and fearing prejudice from teachers (*n* = 43, 46.2%).

Finally, most participants, 512/634 (80.8%), believed they required more information on how to deal with students suffering from bronchial asthma.

### Knowledge of schoolteachers regarding first aid response in acute asthma attack

A majority of participants (445/634, 70.2%) correctly identified the SABA inhaler or nebulizer as appropriate first aid for an acute asthma attack. However, only 187/634 (29.5%) reported knowing the correct technique for using SABA inhalers or nebulizers during an asthma attack. Among those participants (*n* = 187), the most common source of knowledge was a family or relative history of asthma (67.3%), followed by social media (38%). Notably, only five (2.7%) of participants received the information from the teacher preparation program (Table 2).

#### Knowledge evaluation of asthma triggers

Evaluation of knowledge of asthma triggers revealed that 77.8% were aware that chalk dust triggers asthma attacks in students with asthma, whereas dusty weather, passive smoking, and cold weather were recognized by 89.3%, 88.4%, and 69.6%, respectively.

Less than half of the participants, 306/634 (48.3%), thought that food allergies are a common trigger of asthma attacks. Regarding students with bronchial asthma and physical education class, 378 (59.6%) of participants believed that students with asthma are allowed to participate in physical education while doing warm-up exercises appropriate to their health condition, and only 171 (27%) assumed that students with asthma are allowed to participate, provided that the student takes his or her medication before class (Table 3).

**Table 3:** Knowledge of school teachers regarding asthma triggers.

Respondents (*n* = 634)		
In your opinion, can chalk dust trigger asthma attacks in students with asthma? *n* (%)	Yes	493 (77.8%)
	No	22 (3.5%)
	Don’t Know	119 (18.7%)
In your opinion, can dusty weather trigger asthma attacks in students with asthma? *n* (%)	Yes	566 (89.3%)
	No	5 (0.8%)
	Don’t Know	63 (9.9%)
In your opinion, can passive smoking trigger asthma attacks in students with asthma? *n* (%)	Yes	561 (88.4%)
	No	12 (2%)
	Don’t Know	61 (9.6%)
In your opinion, can cold weather in the winter trigger asthma attacks in students with asthma? *n* (%)	Yes	441 (69.6%)
	No	54 (8.5%)
	Don’t Know	139 (21.9%)
Do you think that food allergies are a common trigger of asthma attacks? *n* (%)	Yes	306 (48.3%)
	No	139 (21.9%)
	Don’t Know	189 (29.8%)
Regarding students with bronchial asthma and physical education class *n* (%)	Students with asthma are not allowed to attend the class	34 (5.4%)
	Students with asthma are allowed to participate in the class while doing warm-up exercises appropriate to their health condition	378 (59.6%)
	Students with asthma are allowed to participate in the class, provided that they take their medication before the class	171 (27%)
	Students with asthma are allowed to participate in the class and are not required to take their medications before the class, but the medications are taken when an attack occurs.	126 (19.9%)
	Don’t know	125 (19.7%)

### Comparison between teachers’ sociodemographic characteristics and their level of asthma knowledge

**Fig. 1:**
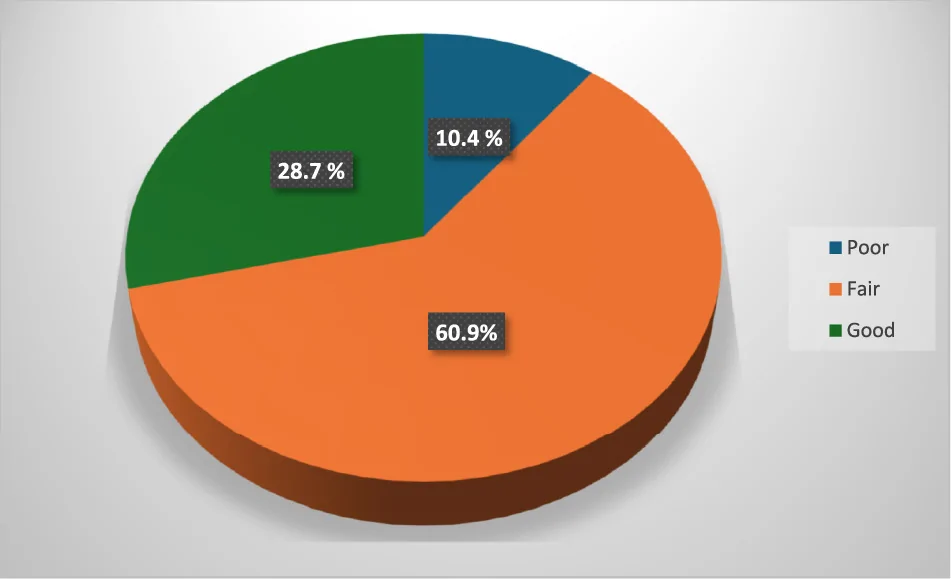
Knowledge score categories and their percent distribution among the studied patricipants. The pie chart demonstrates that 10.4% of participants had poor knowledge, 60.9% had fair knowledge, and 28.7% had good knowledge.

The mean knowledge score of the participants was 19.5 (SD = 2.4), and the mean knowledge score percent was 61% (SD = 8%), with only 10.4% of the teachers showing poor knowledge. Figure 1 shows the categories of the knowledge score of the participants and their percentage distribution. There was no statistically significant association between teachers’ knowledge level and their sociodemographic characteristics, including age (p = 0.09), gender (p = 0.20), years of experience (p = 0.948), school type (p = 0.675), family history of asthma (p = 0.131), number of students previously taught with asthma (p = 0.349), and prior witnessing of an asthma attack (p = 0.847) (Table 4). The multivariable linear regression model was not statistically significant (F = 1.312, p = 0.242), explaining only 1.2% of the variance in knowledge score (R² = 0.012) (Table 5).

**Table 4:** Comparison between teachers’ sociodemographic characteristics and their level of asthma knowledge.

		Knowledge score categories			p-value
		Poor (*n* = 66)	Fair (*n* = 386)	Good (*n* = 182)	
Age (mean ± SD)		42 ± 11	39 ± 10	41 ± 10	0.09
Gender *n* (%)	Male	20 (14.4)	82 (59)	37 (26.6)	0.20
	Female	46 (9.3)	304 (61.4)	145 (29.3)	
Years of experience *n* (%)	1- 4 years	12 (10.4)	74 (64.3)	29 (25.2)	0.948
	5- 9 years	11 (10.7)	62 (60.2)	30 (29.1)	
	10- 14 years	13 (9.5)	88 (64.2)	36 (26.3)	
	15- 19 years	6 (8.8)	41 (60.3)	21 (30.9)	
	≥ 20 years	24 (11.4)	121 (57.3)	66 (31.3)	
School type *n* (%)	Public	35 (11.5)	182 (59.7)	88 (28.9)	0.675
	Private	31 (9.4)	204 (62.0)	94 (28.6)	
Family or relative history of asthma *n* (%)	Yes	20 (7.7)	160 (61.3)	81 (31)	0.131
	No	46 (12.3)	226 (60.6)	101 (27.1)	
How many students have you dealt with who suffered from bronchial asthma? *n* (%)	< 5 students	19 (8.4)	147 (65.3)	59 (26.2)	0.349
	5–10 students	11 (14.5)	39 (51.3)	26 (34.2)	
	> 10 students	5 (8.3)	40 (66.7)	15 (25)	
	I have not dealt with students suffering from bronchial asthma	31 (11.4)	160 (58.6)	82 (30)	
Have you ever witnessed a student having an acute asthma attack? *n* (%)	Yes	19 (10.8)	104 (59.1)	53 (30.1)	0.847
	No	47 (10.3)	282 (61.6)	129 (28.2)	

*SD* standard deviation, p-value is significant <0.05.

**Table 5:** Multiple linear regression addressing predictors for the level of asthma knowledge among participants.

Predictor	Beta	95% CI		P-value
Age	−0.398	−0.037	0.024	0.691
Gender	1.429	−0.131	0.834	0.154
Years of teaching experience	1.511	−0.049	0.375	0.131
School type	−0.059	−0.439	0.413	0.953
Family or relative history of asthma	−1.369	−0.691	0.123	0.171
How many students have you dealt with who suffered from bronchial asthma?	0.800	−0.087	0.207	0.424
Have you ever witnessed a student having an acute asthma attack?	−0.592	−0.595	0.319	0.554
Constant	21.160	17.352	20.902	<0.001

*CI* confidence interval, p-value is significant <0.05.

## Discussion

Bronchial asthma continues to be a substantial public health issue, particularly among school-aged children. Schoolteachers are frequently the first responders during asthma attacks; this is why they play a crucial role in the early identification of symptoms and the provision of first aid. Consequently, the health outcomes of students can be directly influenced by teachers’ knowledge and awareness. The purpose of this study was to address knowledge and awareness gaps among schoolteachers regarding students with asthma in Greater Cairo.

Among the participants who replied to the present questionnaire, 43% of teachers reported never having dealt with asthmatic students despite years of teaching experience, and only 27.8% had witnessed an acute asthma attack previously. This finding highlights a critical discrepancy between the prevalence of asthma in children and the experiential exposure of teachers, which may contribute to under-recognition and under-response to asthma episodes. Such findings emphasize the potentially fatal consequences of unrecognized or mismanaged asthma attacks. Therefore, the application of health education programs about asthma and its attacks is mandatory to increase teachers’ knowledge base. This suboptimal knowledge of asthma among teachers was also noted in many studies assessing schoolteachers’ knowledge regarding asthma across multiple countries such as Spain, the United States, Nigeria, Brazil, Jordan, Saudi Arabia, and Egypt^14,16–20^.

Although 70% of participants correctly identified short-acting beta-agonist inhalers or nebulizers as the appropriate first-line treatment for acute asthma exacerbations, 41% demonstrated inadequate knowledge regarding criteria for hospital transfer, and the majority lacked proficiency in proper administration techniques. Additionally, 43.7% did not know the response time to the inhaler; this deficiency is consistent with several international studies reporting insufficient knowledge among schoolteachers regarding asthma first aid^19,21–23^. These findings suggest training gaps exist in teacher preparation programs and that these programs should not only increase awareness of asthma attacks but also provide classroom teachers with comprehensive training on the proper administration of rescue medications before hospital transfer.

In the present study, the majority of schoolteachers demonstrated better knowledge of the disease’s etiology and nature; for example, only 11.8% of participating schoolteachers believe that bronchial asthma is contagious. In an early study conducted by Aqeel et al., 50.8% of teachers believed that asthma is a contagious disease^24^. Subsequent studies by Karadağ et al. and Awadalla et al. revealed a decline in this misconception, which is consistent with the present study’s findings^20,25^.

Of the teachers, 46.5% associated asthma with poor academic performance, a perception consistent with Plaza-González et al.^26^. Frequent absenteeism due to poorly controlled asthma likely contributes to reduced scholastic achievement, reinforcing the importance of proper asthma management within the educational setting.

A significant proportion of the teachers in this study were aware of potential triggers of asthma attacks, although there were misconceptions and knowledge gaps regarding physical education class as an asthma trigger and how to handle them. This finding was also reported by McClelland et al.^16^. Addressing information gaps about treatment options, including pre-exercise medications, through successful educational initiatives is critical. This would allow students with asthma to continue to engage in physical education class with minimal respiratory symptoms^27^.

Of the respondents, 48.3% believe that food allergies are a common trigger of asthma attacks, although there is a complicated and subtle link between food allergies and asthma. Neither condition causes the other. Foods are seldom the source of isolated acute or chronic asthma symptoms, but lower respiratory symptoms can occur during an acute food allergy reaction^28^.

In view of the present findings, teacher training initiatives and awareness campaigns should be carried out to clarify misconceptions about asthma and increase understanding of the condition to provide more supportive learning environments. Given that 80.2% of respondents were keen to learn more about asthma, we hope to accomplish this goal in Egypt.

Several studies have demonstrated the effectiveness of training programs designed to enhance teachers’ awareness of asthma. Findings consistently revealed a statistically significant improvement in teachers’ knowledge post-intervention compared to pre-intervention assessments^14,29–31^.

These results underscore the necessity of developing and implementing similar awareness programs for teachers across diverse educational settings, using innovative approaches that improve asthma-related knowledge and dispel prevalent myths^32^.

It is worth mentioning that this study has some strengths: it addressed a relevant public health topic and highlighted the implications of school health programs for teachers. The study was conducted in Greater Cairo Governorate, providing a large and diverse sample of socioeconomic and educational environments. It included both public and private schools, allowing for broader representation.

It is also important to recognize several study limitations. First, although the questionnaire underwent forward and back translation, expert content validity assessment, pilot testing, and demonstrated acceptable internal consistency (Cronbach’s α = 0.753), it did not undergo comprehensive psychometric validation, such as construct validity testing or test-retest reliability assessment. Therefore, caution is warranted when comparing the findings with those obtained using fully validated instruments. Second, the use of convenience sampling and online distribution may introduce selection bias, as participation was voluntary and may have favored teachers with a greater interest or awareness of asthma. Third, the reliance on smartphone-mediated communication platforms like WhatsApp constitutes a barrier that excludes teachers lacking digital access. Fourth, causal inference is lacking due to the cross-sectional design of the study.

## Conclusion

The knowledge and awareness of schoolteachers in the Greater Cairo Governorate toward asthma are relatively fair. However, they have notable knowledge gaps, particularly regarding first aid responses. Addressing these gaps through organized teacher preparation programs could significantly improve student outcomes.

## Supplementary information

STROBE Statement—Checklist

## Data Availability

The datasets used and/or analyzed during the current study are available from the corresponding author upon reasonable request.

## References

[1] Masoli, M., Fabian, D., Holt, S., Beasley, R. & Global Initiative for Asthma (GINA) Program. The global burden of asthma: executive summary of the GINA Dissemination Committee report. *Allergy* **59**, 469–478 (2004). 10.1111/j.1398-9995.2004.00526.x 15080825

[2] Alkhamis, Z. N. & Hashim, S. A. Awareness of asthma and its management in primary school teachers in Eastern Province. *J. Family Med. Prim. Care* **8**, 1908–1913 (2019). 10.4103/jfmpc.jfmpc_358_19 PMC6618238 31334154

[3] Canitez, Y., Cekic, S., Celik, U., Kocak, A. & Sapan, N. Health-care conditions in elementary schools and teachers’ knowledge of childhood asthma. *Pediatrics Int. Child. Health* **36**, 64–71 (2016). 10.1179/2046905514Y.0000000150 25203843

[4] Smedje, G. & Norbäck, D. Incidence of asthma diagnosis and self-reported allergy in relation to the school environment—a four-year follow-up study in schoolchildren. *Int. J. Tuberculosis Lung Dis.* **5**, 1059–1066 (2001). 11716342

[5] Hughes, D. Childhood asthma and school. *Paediatrics Child Health* **26**, e4–e5 (2021). 10.1093/pch/pxaa004 PMC7850265 33552324

[6] Global Initiative for Asthma (GINA), 2026. Global strategy for asthma management and prevention. Available at: https://ginasthma.org.

[7] Centers for Disease Control and Prevention, 2017. Strategies for addressing asthma in schools [online].

[8] Doğruel, D. et al. The trend of change of allergic diseases over the years: three repeated surveys from 1994 to 2014. *Int. Arch. Allergy Immunol.* **173**, 178–182 (2017). 10.1159/000477726 28787739

[9] Ahmad, E. E., Kamel, A. S., Amin, A. S. & Hashem, A. E. Epidemiology of childhood asthma in Fayoum City (District) Egypt. *Pharm. Biomed. Sci. J.* **4**, 67–75 (2016).

[10] Georgy, V., Fahim, H. I., El‐Gaafary, M. & Walters, S. Prevalence and socioeconomic associations of asthma and allergic rhinitis in Northern Africa. *Eur. Respir. J.* **28**, 756–762 (2006). 10.1183/09031936.06.00089005 16737986

[11] Dawoud, W., El Kenawy, A. M., Abdel Wahab, M. M. & Oraby, A. H. Temporal variability of particulate matter and black carbon concentrations over Greater Cairo and its atmospheric drivers. *Climate* **11**, 133 (2023).

[12] Ewiss, M. Z. The availability and quality of school buildings in Egypt. *Artic. Int. J. Of. Humanities And. Soc. Sci*. **9**, 56–72 (2020).

[13] Daniel, W. W., 2009. BIOSTATISTICS: A Foundation for Analysis in the health sciences 9TH ED. Wiley.

[14] Urturi, A. F., Puertas, R. A., Romero, A. C., del Amo Ramos, S. & Fernández, M. S. Impact of an educational intervention about asthma on early childhood and primary education teachers in one health district. *Rev. Pediatr.* **22**, 353–360 (2020).

[15] Kuyinu, Y. A., Adeyeye, O. O. & Ozoh, O. B. Assessment of the knowledge of teachers about asthma and the availability of facilities for asthma care in public secondary schools in Lagos, Nigeria. *Afr. J. Thorac. Crit. Care Med.* **24**, 76–81 (2018). 10.7196/AJTCCM.2018.v24i2.192 PMC8432919 34541499

[16] McClelland, Q. Y. L., Avalos, M. I. & Reznik, M. Asthma management in New York City schools: A physical education teacher perspective. *J. Asthma* **56**, 422–430 (2019). 10.1080/02770903.2018.1463380 PMC6295268 29667459

[17] Ibraheem, R. M., Hamzah, A. L. O., Ameen, H. A., Katibi, O. S. & Johnson, A. W. R. Evaluation of the awareness and experiences of the primary and secondary school teachers on asthma: a cross-sectional study in Ilorin, Nigeria. *J. Pan Afr. Thorac. Soc.* **3**, 140–150 (2022).

[18] Brosso, L. et al. Knowledge and experience of Primary Education teachers regarding childhood asthma: mixed study. *Rev. da Esc. de. Enferm. da USP.* **57**, e20220329 (2023). 10.1590/1980-220X-REEUSP-2022-0329en PMC10205126 37216653

[19] Nour, A., Alsayed, A. R. and Basheti, I., 2023, January. Prevalence of asthma amongst schoolchildren in Jordan and staff readiness to help. In Healthcare (Vol. 11, p. 183). MDPI. 10.3390/healthcare11020183 PMC9859049 36673551

[20] Awadalla, M. F. M. et al. Knowledge and response of school teachers towards asthma exacerbation among school children in two arab countries. *Clin. Epidemiol. Glob. Health* **26**, 101533 (2024).

[21] Kovačič, U., Lozić, A., Slabe, D. & Starc, A. The knowledge of teachers as a key factor in providing first aid in primary schools. *J. Appl. Health Sci.= Časopis za primijenjene zdravstvene znanosti* **5**, 17–29 (2019).

[22] Chakravarthy, D., Sankaranarayanan, S. & David, A. Evaluating the impact of a teaching program on knowledge, quality of life, and symptoms among asthmatic children. *Cureus* **17**, e81022 (2025). 10.7759/cureus.81022 PMC12012592 40264615

[23] Hidayah, I. N. & Ramdani, M. L. The impact of asthma management health education on asthma knowledge and first aid skills. *Proc. Ser. Health Med. Sci.* **6**, 146–151 (2025).

[24] Aqeel, T., Akbar, N., Dhingra, S. & Haq, N. U. Assessment of knowledge and awareness regarding asthma among school teachers in the urban area of Quetta, Pakistan. *J. Pharm. Pract. Community Med.* **1**, 18–23 (2015).

[25] Karadağ, M., Macunluoğlu, A. C., Demirdöğen Çetinoğlu, E. & Ediger, D. Evaluation of asthma awareness among teachers in Bursa. *Tuberk. ve Toraks* **66**, 150–155 (2018). 10.5578/tt.67040 30246659

[26] Plaza-González, S., Zabala-Baños, M. D. C., Astasio-Picado, Á. & Jurado-Palomo, J. Psychological and sociocultural determinants in childhood asthma disease: impact on quality of life. *Int. J. Environ. Res. Public. Health* **19**, 2652 (2022). 10.3390/ijerph19052652 PMC8909745 35270342

[27] Parsons, J. P. et al. An official American Thoracic Society clinical practice guideline: exercise-induced bronchoconstriction. *Am. J. Respir. Crit. Care Med.* **187**, 1016–1027 (2013). 10.1164/rccm.201303-0437ST 23634861

[28] Stukus, D. R. & Prince, B. T. Asthma and food allergy: A nuanced relationship. *J. Food Allergy* **5**, 33–37 (2023). 10.2500/jfa.2023.5.230009 PMC11250531 39022752

[29] Neuharth-Pritchett, S. & Getch, Y. Q. The effectiveness of a brief asthma education intervention for child care providers and primary school teachers. *Early Child. Educ. J.* **44**, 555–561 (2016).

[30] ES, A. G. & El-Herishi, S. Asthma education for school staff in Riyadh city: effectiveness of pamphlets as an educational tool. *J. Egypt. Public. Health Assoc.* **82**, 147–171 (2007). 18217329

[31] Henry, R. L., Gibson, P. G., Vimpani, G. V., Francis, J. L. & Hazell, J. Randomized controlled trial of a teacher‐led asthma education program. *Pediatric Pulmonol.* **38**, 434–442 (2004). 10.1002/ppul.20095 15690558

[32] Naidoo, K. L. et al. A cross-country qualitative analysis of teachers’ perceptions of asthma care in sub-Saharan Africa. *NPJ Prim. Care Respir. Med.* **33**, 31 (2023). 10.1038/s41533-023-00354-7 PMC10517916 37741822

